# S-NER: A Concise and Efficient Span-Based Model for Named Entity Recognition

**DOI:** 10.3390/s22082852

**Published:** 2022-04-08

**Authors:** Jie Yu, Bin Ji, Shasha Li, Jun Ma, Huijun Liu, Hao Xu

**Affiliations:** College of Computer, National University of Defense Technology, Changsha 410073, China; yj@nudt.edu.cn (J.Y.); jibin@nudt.edu.cn (B.J.); majun@nudt.edu.cn (J.M.); liuhuijun@nudt.edu.cn (H.L.); xuhao@nudt.edu.cn (H.X.)

**Keywords:** named entity recognition, span-based model, BERT

## Abstract

Named entity recognition (NER) is a task that seeks to recognize entities in raw texts and is a precondition for a series of downstream NLP tasks. Traditionally, prior NER models use the sequence labeling mechanism which requires label dependency captured by the conditional random fields (CRFs). However, these models are prone to cascade label misclassifications since a misclassified label results in incorrect label dependency, and so some following labels may also be misclassified. To address the above issue, we propose S-NER, a span-based NER model. To be specific, S-NER first splits raw texts into text spans and regards them as candidate entities; it then directly obtains the types of spans by conducting entity type classifications on span semantic representations, which eliminates the requirement for label dependency. Moreover, S-NER has a concise neural architecture in which it directly uses BERT as its encoder and a feed-forward network as its decoder. We evaluate S-NER on several benchmark datasets across three domains. Experimental results demonstrate that S-NER consistently outperforms the strongest baselines in terms of F1-score. Extensive analyses further confirm the efficacy of S-NER.

## 1. Introduction

Named entity recognition (NER) is a crucial subtask of information extraction. It is taken as an essential prerequisite for many other natural language processing (NLP) tasks [1] such as relation extraction, question answering and co-reference resolution.

**Figure 1:**
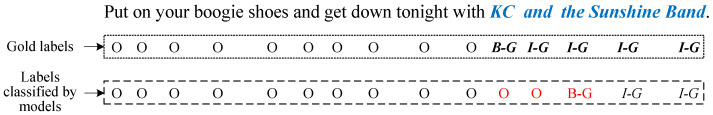
An example of sequence labeling-based NER. The “*KC and the Sunshine Band*” (in blue font) is a pre-defined Group entity, and its gold BIO label sequence is {B-G,I-G,I-G,I-G,I-G}. However, the sequence labeling-based NER model actually tags the “*KC*” with the O label, resulting in cascading label misclassifications, i.e., the O label for “*and*”, the B-G label for “*the*”, as the labels in red show.

The majority of neural NER models use the sequence labeling mechanism [2,3,4]. In this paper, we regard these NER models to be multi-class classification models since they tag each text token with one BIO label through multi-class label classifications. These NER models make attempts to improve their performance by employing complicated model encoders but they use almost the same model decoder: a feed-forward network (FFN) followed by the conditional random fields (CRFs), where the CRF captures label dependency that is used to ensure the label consistency [5,6]. Since the decoders sequentially conduct label classifications, these models may suffer from cascading label misclassifications since once a label is misclassified, CRF would capture wrong label dependency. As shown in Figure 1, the model decoder actually classifies the “*KC*” into the O label, which is supposedly classified into the B-G label. As a result, the decoder misclassifies two subsequent labels (i.e., the O label for “*and*” and the B-G label for “*the*”) due to the wrong label dependency.

The span-based model is appropriate for resolving the above problem since it completely abstains from the sequence labeling mechanism which eliminates the requirement for label dependency. Previous span-based NER models [1,7,8,9] are proposed to recognize nested entities by regarding text spans as candidate entities where spans can be nested. Text spans are continuous text segments of which the length is restricted by a length threshold ϵ [10,11]. Formally, given a text of $T=(t_{1},t_{2},\ldots ,t_{n})$ and the ϵ value, all possible spans can be denoted by s=(a,b), where *a* and *b* are indices of span head and tail tokens, 1≤a≤b≤n and (b−a+1)≤ϵ. Consider the following text: “It${}_{1}$ snows${}_{2}$ today${}_{3}$ .${}_{4}$”. Here, when setting the ϵ to 3, all possible spans are {(1, 1), (2, 2), (3, 3), (4, 4),(1, 2), (2, 3), (3, 4), (1, 3), (2, 4)}. For example, the s = (2, 2) denotes “snow” and the s = (2, 3) denotes “snow today”. We can observe that spans are allowed to be nested, such as (2, 2) and (2, 3), which enables the model to recognize nested entities. However, to the best of our knowledge, no span-based NER model is proposed to investigate its effects in tackling the cascading label misclassification problem.

To solve the above problem, we propose a concise and efficient span-based model which we refer to as S-NER. In contrast to the existing span-based NER models [9,12] that usually combine BERT and complicated neural architectures as the model decoders, S-NER directly utilizes BERT as its encoder and a feed-forward network (FFN) as its decoder. When recognizing entities, S-NER first splits raw texts into text spans; it then obtains span semantic representations by concatenating span representations, contextual representations and span length embeddings, where the first two are generated by BERT and the last one is trained during model training; finally, it obtains span types by conducting entity type classifications on span semantic representations.

Previous span-based NER models actually enumerate all possible spans during model training, leading to low training efficiency. In this paper, we demonstrate that a sufficient number of randomly sampled negative spans are sufficient to ensure good model performance which greatly improves the training efficiency. More details can be found in Section 4.4. Additionally, we investigate multiple methods to obtain span semantic representations, including max-pooling, average-pooling and boundary embedding. More details can be found in Section 4.6.

To evaluate S-NER, we conduct experiments on several benchmark datasets from three domains, i.e., news, social and scientific. Experimental results demonstrate that S-NER consistently outperforms the previous best models on the chosen benchmarks in terms of F1-score, despite the fact that some of these models employ the multi-task learning paradigm or make use of additional data resources to improve their performance. Moreover, we analyze the model performance against cascading label misclassifications in Section 4.3 and report a detailed case study in Section 4.8. Furthermore, we undertake the rigorous trials to further confirm the efficiency of S-NER.

In summary, the contributions of this paper can be concluded as follows: (1) we propose a span-based model that aims to address the cascading label misclassification problem existing in sequence labeling-based NER models; (2) our model is concise in the neural architecture, and explores using a negative sampling strategy to improve the model training efficiency; (3) experimental results on three benchmark datasets demonstrate that S-NER creates new state-of-the-art results in terms of F1-score and our model provides additional macro-average F1-score on these benchmarks.

## 2. Related Work

### 2.1. Sequence Labeling-Based NER Model

Prevalent neural models for NER use the sequence labeling mechanism. These models are mainly concerned with the design of novel neural architectures as their encoders, such as recurrent neural network (RNN) [13,14], convolutional neural network (CNN) [15,16] and transformer encoder [4,17]. Recently, pre-trained language models, such as BERT [18] and RoBERTa [19], are applied to this research field, which are directly used as model encoders. However, these models require label dependency, which in some cases causes cascading label misclassifications. Compared to these models, our model uses the span-based paradigm and no longer calls for the label dependency.

### 2.2. Span-Based NER Model

Recently, a few span-based NER models have been proposed to recognize nested entities. Fu et al. [20] proposed a span-based NER model, which combines BiLSTM [21] and BERT [18] as the embedding layer. The model obtains span semantic representations using the boundary embedding method and considers all possible spans. Li et al. [9] proposed a similar span-based NER model, while they use an attention-guided graph convolutional network (AGGCN) [22] to incorporate the dependency syntax information into word embeddings. Moreover, the model enables them to double-check nested entities and detect discontinuous entities. Tan et al. [7] proposed to jointly train a span-based NER model and an entity boundary detection model. The boundary and span classification results jointly determine whether a span is an entity. Moreover, Ouchi et al. [12] formulated the nested NER task as an instance-based learning problem. They obtain span types through the best similarity between test spans and entities in the training set. Yu et al. [23] formulated the task as a dependency parsing problem. They used a biaffine model [24] to predict the types for all spans of a given text, where the spans are restricted by head-child positions. In contrast to the above models, our model is proposed to investigate its effects against the cascading label misclassification problem. Moreover, we propose using sampled spans instead of all possible spans, which greatly improves model training efficiency.

### 2.3. Span-Based Model for Joint Entity and Relation Extraction

Span-based models have been comprehensively investigated for the task of joint entity and relation extraction. Luan et al. [25] proposed the first span-based model for the task. Subsequently, Dixit and Al-Onaizan [11] proposed a span-based joint model, which first obtains span semantic representations through the BiLSTM and ELMo [26] and then shares them in both span and relation classifications. Following Luan et al. [25], Luan et al. [10] proposed DyGIE, which can capture span interactions through a dynamically constructed span graph. Wadden et al. [27] improved DyGIE by replacing the BiLSTM with BERT and propose DyGIE++. More recently, Eberts and Ulges [28] proposed SpERT, which takes BERT as the backbone. However, the above span-based models generally use carefully designed model decoders, and are proposed for the joint extraction task. Compared to them, our model adopts a concise neural architecture and is specialized in NER.

## 3. Model

In this section, we first illustrate the overall architecture of S-NER and then introduce the model encoder (Section 3.1), the model decoder (Section 3.2), and the negative sampling strategy (Section 3.3), respectively.

**Figure 2:**
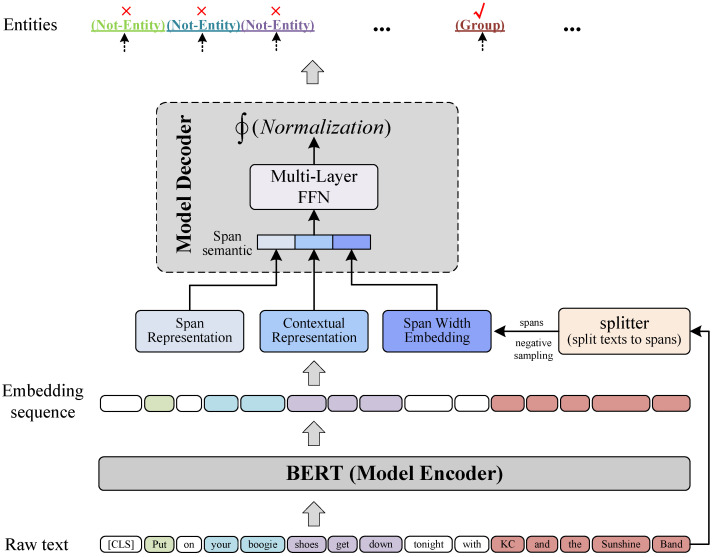
Overall view of S-NER. Text tokens with the same background color (except white) are span examples, such as “Put”, “your boogie”, “shoes get down”, “KC and the Sunshine Band”.

As shown in Figure 2, S-NER is composed of a model encoder and a model decoder. It directly takes BERT as its model encoder, and the model decoder is a multi-layer FFN followed by a normalization function. For a given raw text, the encoder first generates its BERT embedding sequence; then, the splitter module splits the text into text spans and obtains span semantic representations; at last, the decoder obtains span types by conducting entity type classifications on span semantic representations.

It is worth noting that we add a Not-Entity type to the pre-defined entity types and we assign the Not-Entity type to spans that are not entities.

### 3.1. Model Encoder

The model encoder is essentially a BERT model. Given a raw text $T=\{t_{1},t_{2},t_{3},\ldots ,t_{n}\}$ where $t_{i}$ denotes the *i*-th token of the text, we first add a specific (CLS) token to the beginning and denote the T as follows (The addition of (CLS) is required by BERT):

(1) $$T=\left\{t_{0},t_{1},t_{2},t_{3},\ldots ,t_{n}\right\}\;s.t.\;t_{0}=\left[\mathrm{CLS}\right]$$

For T, BERT first tokenizes it with the WordPiece vocabulary [29] to obtain an input sequence $T^{\prime }=\left\{t_{0}^{\prime },t_{1}^{\prime },t_{2}^{\prime },\ldots ,t_{m}^{\prime }\right\}$. For each $t_{i}^{\prime }$ of $T^{\prime }$, its representation is the element-wise addition of WordPiece embedding, positional embedding, and segment embedding. Then, a list of input embeddings $H\in R^{(m+1)*h}$ are obtained, where (m+1) is the length of the input sequence and *h* is the size of hidden units. A series of pre-trained transformer blocks [30] are then used to project H to the BERT embedding sequence (denoted as E):

(2) $$E=\{e_{0},e_{1},e_{2},e_{3},\ldots ,e_{m}\}$$

where $e_{i}\in R^{d}\;\left(0\leq i\leq m\right)$ and *d* is the BERT embedding dimension.

BERT may tokenize a token into several sub-tokens to alleviate the out-of-vocabulary (OOV) problem, leading to T being unable to align with E, i.e., m≠n. To solve this problem, for each token $t_{i}$ in T, we apply the max-pooling function to the BERT embeddings of its sub-tokens to obtain its BERT embedding. We denote the aligned BERT embedding sequence of T as follows:

(3) $$E^{\prime }=\left\{{\hat{e}}_{0},{\hat{e}}_{1},{\hat{e}}_{2},{\hat{e}}_{3},\ldots ,{\hat{e}}_{n}\right\}$$

where ${\hat{e}}_{i}\in R^{d}$ is the BERT embedding of the token $t_{i}$ in T.

Then, the splitter module splits T into text spans. Text spans are continuous text segments the length of which is restricted by a length threshold ϵ. Given the text “It snows today .” and ϵ=3, we denote all possible spans as follows:

(4) $$\mathrm{spans}=\left\{\begin{array}{c} \mathrm{span}\;\mathrm{length}=1:\{“\mathrm{It}”,\;“\mathrm{snows}”,\;“\mathrm{today}”,\;“.”\}\\ \mathrm{span}\;\mathrm{length}=2:\{“\mathrm{It}\;\mathrm{snows}”,\;“\mathrm{snows}\;\mathrm{today}”\;,“\mathrm{today}\;.”\}\\ \mathrm{span}\;\mathrm{length}=3:\{“\mathrm{It}\;\mathrm{snows}\;\mathrm{today}”,\;“\mathrm{snows}\;\mathrm{today}\;.”\} \end{array}\right.$$

Thus, for the given text T and the ϵ value, we formulate its spans as follows:

(5) $$s=\{t_{i},t_{i+1},\ldots ,t_{i+j}\}\;s.t.\;1\leq i\leq i+j\leq n,\;\;j<\epsilon$$

where the added (CLS) token are not taken into account and a total number of $\frac{\epsilon *(2n-\epsilon +1)}{2}$ spans can be obtained. We denote the BERT embedding sequence for *s* as follows, which is derived from $E^{\prime }$:

(6) $$E_{s}=\left\{{\hat{e}}_{i},{\hat{e}}_{i+1},\ldots ,{\hat{e}}_{i+j}\right\}$$

We then obtain the span semantic representation by concatenating span representation (Section 3.1.1), contextual representation (Section 3.1.2) and span length embedding (Section 3.1.3).

#### 3.1.1. Span Representation

For span *s*, we obtains its representation by applying the max-pooling function to its BERT embedding sequence $E_{s}$:

(7) $$R_{s}=\left[max\left({\hat{e}}_{i,1},{\hat{e}}_{i+1,1},\ldots ,{\hat{e}}_{i+j,1}\right),max\left({\hat{e}}_{i,2},{\hat{e}}_{i+1,2},\ldots ,{\hat{e}}_{i+j,2}\right),\ldots ,max\left({\hat{e}}_{i,d},{\hat{e}}_{i+1,d},\ldots ,{\hat{e}}_{i+j,d}\right)\right]$$

In Section 4.5, we compare various methods to obtain the span representation, including max-pooling, average-pooling, and boundary embedding. The comparison results demonstrate that the max-pooling method is the best.

#### 3.1.2. Contextual Representation

In this paper, we take the BERT embedding of (CLS) (i.e., ${\hat{e}}_{0}$) as the contextual representation for any *s* in T, which follows Ji et al. [31] and Luan et al. [10].

#### 3.1.3. Span Length Embedding

Span length embedding allows the model to incorporate prior experience over span lengths. In this paper, we train fixed-size embeddings for each span length (i.e., 1, 2, …, ϵ) during model training. Furthermore, we denote the length embedding for *s* (length is *j*+1) as $W_{j+1}$.

#### 3.1.4. Span Semantic Representation

Finally, we concatenate the above three representations to obtain the span semantic representation, as shown below:

(8) $${\hat{E}}_{s}=\left[R_{s};{\hat{e}}_{0};W_{j+1}\right]$$

### 3.2. Model Decoder

In this paper, we denote the set of pre-defined entity types and the Not-Entity as Φ. The model decoder obtains span types by conducting entity type classifications on span semantic representations. As shown in Figure 2, the decoder is a multi-layer FFN which is followed by a normalization function.

The multi-layer FFN deep stacks multiple FFNs, and is used to convert ${\hat{E}}_{s}$ from the embedding space of span semantic representations to the embedding space of Φ:

(9) $${\hat{E}}_{s}^{\prime }=W{\hat{E}}_{s}+b$$

where ${\hat{E}}_{s}^{\prime }\in R^{\left|\Phi \right|}$ and |Φ| is the count of entity types in Φ. W and b are FFN parameters. Then, ${\hat{E}}_{s}^{\prime }$ is passed to the normalization function, which yields a posterior for *s* on Φ. In this paper, we use the softmax function as the Normalization function:

(10) $${\hat{y}}_{s,i}=\frac{\exp^{{\hat{E}}_{s,i}^{\prime }}}{\sum_{j=1}^{\left|\Phi \right|}\exp^{{\hat{E}}_{s,j}^{\prime }}}\;s.t.\;1\leq i\leq \left|\Phi \right|$$

The highest response in ${\hat{y}}_{s}$ indicates that the corresponding entity type is considered activated. The training objective is to minimize the following cross-entropy loss:

(11) $$L=-\frac{1}{N}\sum_{k=1}^{N}\sum_{i=1}^{\left|\Phi \right|}y_{k,i}\log{\hat{y}}_{k,i}$$

where $y_{k}$ is the one-hot vector of gold span type and *N* is the number of span instances.

### 3.3. Negative Sampling Strategy

Previous span-based NER models [1,9,20] use all possible spans during model training, which results in lower model training efficiency. For example, if the length (*n*) of the text T is 50 and the span length threshold ϵ is set to 10, a total of 455 spans will be generated, which is calculated by $\frac{\epsilon *(2n-\epsilon +1)}{2}$. Motivated by the fact that a sufficient number of negative spans suffice to ensure good model performance [28], we propose to sample a small number of negative spans (i.e., spans of the Not-Entity type) during model training. To be specific, we randomly sample a max number of δ negative spans for each text in the training set. Then, we combine the sampled negative spans and the gold entities for model training. Considering the fact that the count of all possible spans (i.e., $\frac{\epsilon *(2n-\epsilon +1)}{2}$) is less than δ in some cases, we actually sample a number of min($\frac{\epsilon *(2n-\epsilon +1)}{2}$, δ) negative spans for the text T.

During model inference, we predict the types of all possible spans of test texts.

## 4. Experiment

### 4.1. Experimental Setup

#### 4.1.1. Datasets

We evaluate S-NER on the three benchmark datasets from three domains, namely WNUT2016 [32] (social), CoNLL2004 [33] (news) and SciERC [25] (scientific).

- The WNUT2016 dataset is constructed from Twitter. It includes ten types of entity (i.e., Geo_Loc, Facility, Movie, Company, Product, Person, Other, Sportsteam, TVShow and Musicartist). We use the same training (2934 sentences), development (3850 sentences) and test set (1000 sentences) split proposed by Nie et al. [34].
- The CoNLL2004 dataset consists of sentences from news articles. It includes four types of entity (i.e., People, Location, Organization and Other) and five types of relation (i.e., Work-For, Live-In, Kill, Organization-Based-In and Located-In). In this paper, we only use the entity annotations. We use the same training (1153 sentences) and test set (288 sentences) split proposed by Eberts and Ulges [28]. Moreover, 20% of the training set is taken as a held-out development part for hyperparameter tuning.
- The SciERC dataset is derived from 500 abstracts of AI papers and is composed of a total of 2687 sentences. It includes six types of scientific entity (i.e., Task, Material, Other-Scientific-Term, Method, Metric and Generic) and seven types of relation (Compare, Feature-Of, Part-Of, Conjunction, Evaluate-For, Used-For and Hyponym-Of). We only use the entity annotations. Moreover, we use the same training (1861 sentences), development (275 sentences) and test (551 sentences) split used by Eberts and Ulges [28].

More details about the above benchmark datasets could be found in the paper [28,34].

#### 4.1.2. Implementation Details

For all datasets, we evaluate S-NER with the bert-large-cased model on a single NVIDIA RTX 3090 GPU. For a fair comparison with prior work, we also use SciBERT [35] when evaluating S-NER on SciERC. We optimize S-NER using AdamW for 20 epochs with a learning rate of $5\times 10^{-5}$, a linear scheduler with a warm-up ratio of 0.1 and a weight decay of $1\times 10^{-2}$. We set dimensions of $W_{j+1}$ to 25 AND the max negative span count δ to 100. For each experiment, We run our model for 5 runs and report the averaged micro- and macro-average F1-scores to evaluate the model performance.

Moreover, we set the training batch size to 32 for WNUT16 and 8 for CoNLL04 and SciERC, respectively. We set the span length threshold (ϵ) to 6 for WNUT16 and 10 for CoNLL04 and SciERC, ensuring that more than 99.5% of entities can be covered.

### 4.2. Main Results

We report the performance comparison between S-NER and previous best models in Table 1. We can observe that S-NER consistently outperforms the strongest baselines on all benchmarks in terms of F1-score.

Precisely, (1) on WNUT2016, S-NER delivers +1.14% absolute F1 gains when compared to the previous best model CL-KL [39]; (2) on SciERC, S-NER outperforms the previous best model RDANER [42] by up to +0.50% absolute F1-score when not using SciBERT, and surpasses the SpERT +0.22% absolute F1-score when using SciBERT; (3) on CoNLL2004, S-NER outperforms previous best models by up to +0.26% and +1.41% absolute F1-score under micro- and macro-average measures, respectively; (4) S-NER provides the macro-average F1-score on WNUT2016 and SciERC, which can be used for future study.

Additionally, we want to emphasize that all the baselines for CoNLL2004 and the majority of baselines for SciERC adopt the multi-task learning (MTL) paradigm. It is well known that the MTL can boost NER performance by using the information derived from other NLP tasks, which makes it unfair for the performance comparison. Moreover, the two previous best models (CL-KL and RDANER) use extra data resources to promote their performance, which also makes it unfair for the comparison. However, S-NER still consistently outperforms them, demonstrating the effectiveness of S-NER.

### 4.3. Performance against Cascading Label Misclassification

In this section, we compare S-NER with the previous best sequence labeling-based NER models across the three benchmark datasets. Specifically, we use the CL-KL [39] for WNUT2016, and Table-sequence [48] for CoNLL2004 and the RDANER [42] for SciERC. For each benchmark dataset, we keep a subset of its test set where the subset comprises cascading label misclassifications induced by the sequence labeling-based model. To be more precise, we solely choose test sentences in which at least one gold entity is predicted as two wrong entities, as the three cases in Section 4.8 show. Finally, 141, 79 and 105 test sentences are drawn from the test sets of WUNT2016, CoNLL2004 and SciERC, respectively. On the three subsets, we compare the performance of our model and sequence labeling-based models, and report the results in Table 2. We observe that our model significantly improves the performance on the three subsets, i.e., +36.84%, +46.02% and +23.32% F1-scores, indicating that our model is effective at mitigating cascading label misclassifications.

### 4.4. Performance against Negative Sampling Strategy

**Figure 3:**
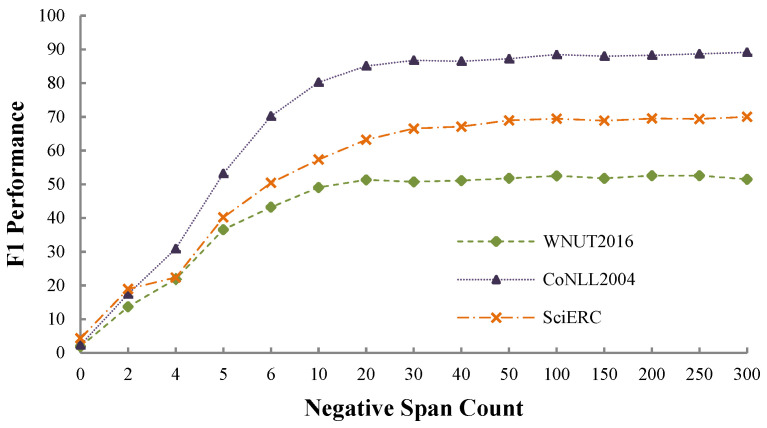
Performance comparisons on the dev sets of the three benchmark datasets when setting the negative span count δ to various values. We set δ to 100 in all other experiments.

We conduct experiments on the dev sets of the three benchmark datasets to investigate the effects of the negative sampling strategy. We set the negative span count δ to different values, i.e., 0, 2, 4, 6, 8, 10, 20, 30, 40, 50, 100, 150, 200, 250 and 300. We report the experimental results in Figure 3, from which we observe that a sufficient number of negative spans is essential to ensure that the model performs well. Specifically, (1) when setting the count to 0, the F1-scores are approximately 1.88% (WNUT2016), 2.35% (CoNLL2004), and 3.44% (SciERC), which are fairly low; (2) when setting the count to a sufficiently large number (such as ≥50), performance across all datasets becomes stagnant. However, we found the F1-score to be more stable when setting δ to 100, which is the default setting for all the other experiments. Moreover, it is a trade-off between model performance and model training and inference efficiency.

To further evaluate the effectiveness of the negative sampling strategy, we compare model performances between S-NER with (δ = 100) and without (using all possible spans) the strategy. We report the model performance tested on the dev sets of the three benchmark datasets, as shown in Table 3. We observe that using all possible spans improves the model performance in the majority of cases, such as on WNUT2016 (+0.4%) and SciERC (+0.59%), but degrades the model performance on CoNLL2004 (−0.24%), indicating that employing all possible spans may not be the ideal decision. Furthermore, it remains a challenge despite our efforts to determine a more appropriate δ value that will result in an improved model performance across the three benchmark datasets.

Additionally, we compare the counts of sampled spans and all possible spans across the three benchmark datasets. We calculated the averaged counts across all training sentences and reported the comparison results in Table 4. We observe that the negative sampling strategy enables our model to train with a significantly reduced number of negative spans: as little as 48.5%, 35.4% and 48.5% of all possible spans are sampled on the WNUT2016, CoNLL2004 and SciERC, respectively.

We suppose that fewer negative spans indicates the faster training of the model. To verify this hypothesis, we compare the model training speeds across the three benchmark datasets which are measured by the number of sentences processed per second, as shown in Table 5. We observe that the negative sampling strategy consistently boosts the speeds. For example, our model trained using sampled spans is twice as fast as the model trained using all possible spans on the CoNLL2004.

### 4.5. Performance against Decoder Layers

To evaluate the model performance against the decoder with various FFN layers, we conduct experiments on the dev sets of the three benchmark datasets and in-depth stack FFN layers if the model decoder contains more than one FFN layer. We report the performance comparisons in Table 6 from which we can observe that (1) S-NER with 1-FFN obtains the best performance on WNUT2016 and SciERC; (2) S-NER with 3-FFNs performs the best on CoNLL2004. As the averaged performance of the former is better than that of the latter (70.00% vs. 69.33%) and the former is more time-saving for model training and inference since fewer parameters are needed, we choose the former as the default decoder setting.

### 4.6. Performance against Span Representation

We adopt three different approaches, i.e., max-pooling, average-pooling and boundary embedding to obtain the span representation. The max-pooling one was elaborated in Section 3.1. We achieve the average-pooling method by applying the average-pooling function to span embedding sequence $E_{s}$, as shown below:

(12) $$R_{s}=\frac{1}{j+1}\left[\sum \left({\hat{e}}_{i,1},{\hat{e}}_{i+1,1},\ldots ,{\hat{e}}_{i+j,1}\right),\sum \left({\hat{e}}_{i,2},{\hat{e}}_{i+1,2},\ldots ,{\hat{e}}_{i+j,2}\right),\ldots ,\sum \left({\hat{e}}_{i,d},{\hat{e}}_{i+1,d},\ldots ,{\hat{e}}_{i+j,d}\right)\right]$$

We achieve the boundary embedding method by concatenating the BERT embeddings of span head and span tail tokens, as shown below:

(13) $$R_{s}=\left[{\hat{e}}_{i};{\hat{e}}_{i+j}\right]$$

We conduct experiments on the dev sets of the three benchmark datasets and report the results in Table 7. We can observe that the max-pooling one achieves the best performance across all three benchmark datasets, which motivates us to use it as the default setting in all other experiments.

### 4.7. Investigation of Model Training Speed

In this section, we compare the model training speed of our model and those of sequence labeling-based NER models. To be specific, we select the CL-KL [39] for WNUT2016, the Table-sequence [48] for CoNLL2004 and the RDANER [42] for SciERC, which are the current best sequence labeling-based models on the three datasets. For a fair comparison, we use an identical training batch size for the two models tested on the same dataset. We report the investigation results in Table 8, which are measured by the number of sentences processed per second. We can observe that our model is consistently trained faster than the three models, delivering 1.22×, 2.09× and 1.43× speedups on the three datasets, respectively. We attribute them to our model being more concise in its neural architecture and we propose the use of sampled negative spans rather all possible spans during model training.

### 4.8. Case Study

Additionally, we give a detailed depiction of typical case studies concerning how S-NER tackles cascading label misclassifications, as shown in Table 9. We have the following observations: (1) the misclassification can lead to severe entity prediction errors—for example, it results in two wrong entities in each of the three cases; (2) S-NER is capable of solving the entity prediction errors caused by the misclassification since the span-based paradigm eliminates the requirements of label dependency.

## 5. Conclusions

In this paper, we propose S-NER, a span-based model aiming to solve the cascading label misclassifications existing in sequence labeling-based NER models. S-NER directly takes BERT as its encoder and an FFN as its decoder, which is concise in model architecture. Experimental results on several benchmark datasets from three domains demonstrate that S-NER outperforms the previous best models in terms of F1-score. Nevertheless, some of these best models promote NER performance by adopting the multi-task learning paradigm or using extra data resources. Moreover, detailed case studies and extra experiments on the model decoder, negative sampling strategy and span representation further validate the effectiveness of S-NER.

## Figures and Tables

**Table 1:** Main results on the test sets of benchmarks. ♠: using extra data resources such as gazetteer. Bold values denote the best results.

Dataset	Model	Multi-Task Learning	F1-Score	
			Micro	Macro
WNUT2016	BERTweet [36]	-	52.10	-
	DATNet [37]	-	53.43	-
	InferNER [38]	-	53.48	-
	TENER [4]	-	54.06	-
	SANER [34]	-	55.01	-
	SpanNER [20]	-	56.27	-
	CL-KL [39] ♠	-	58.98	-
	S-NER	-	**60.12 (+1.14)**	**46.77 (new)**
SciERC	SciE [25]	✓	64.20	-
	SciBERT [35]	-	65.50	-
	DyGIE++ [27]	✓	67.50	-
	SpERT [28]	✓	67.62	-
	PFN [40]	✓	66.80	-
	PURE [41]	✓	68.90	-
	RDANER [42] ♠	-	68.96	-
	SpERT (SciBERT) [28]	✓	70.33	-
	S-NER	-	**69.46 (+0.50)**	**69.47 (new)**
	S-NER (SciBERT)	-	**70.55 (+0.22)**	**70.71 (new)**
CoNLL2004	Multi-head [43]	✓	-	83.90
	Relation-metric [44]	✓	-	84.15
	Biaffine [24]	✓	-	86.20
	Global [45]	✓	85.60	-
	Multi-turn QA [46]	✓	87.80	-
	SpERT [28]	✓	88.94	86.25
	Deeper [47]	✓	89.78	87.00
	Table-sequence [48]	✓	90.10	86.90
	S-NER	-	**90.36 (+0.26)**	**88.41 (+1.41)**

**Table 2:** Performance comparisons on test subsets across the three benchmark datasets, where the sentences of subsets consist of cascading label misclassifications. Bold values denote the best results.

Model	WNUT16 (F1-Score)	Model	CoNLL2004 (F1-Score)	Model	SciERC (F1-Score)
CL-KL	16.79	Table-sequence	27.44	RDANER	21.57
S-NER	**53.63**	S-NER	**73.46**	S-NER	**44.89**

**Table 3:** Performance comparisons under the conditions of using and not using the randomly sampling strategy. We report the performance on the dev sets of the three benchmark datasets. Bold values denote the best results.

Model	WNUT2016 (F1-Score)	CoNLL2004 (F1-Score)	SciERC (F1-Score)
S-NER +			
w negative sampling strategy	53.77	**87.36**	68.87
w/o negative sampling strategy	**54.17**	87.12	**69.46**

**Table 4:** Comparisons of negative span counts per training sentence under the conditions of using and not using the randomly sampling strategy.

Model	WNUT2016 (Spans/Sent)	CoNLL2004 (Spans/Sent)	SciERC (Spans/Sent)
S-NER +			
w negative sampling strategy	81	87	96
w/o negative sampling strategy	167	246	198

**Table 5:** Comparisons of model training speed when using and not using the negative sampling strategy. ↑ denotes the larger the value, the faster the speed.

Model	WNUT2016 (Sents/s ↑)	CoNLL2004 (Sents/s ↑)	SciERC (Sents/s ↑)
S-NER +			
w negative sampling strategy	44	67	53
w/o negative sampling strategy	32	31	34

**Table 6:** Performance against the model decoder with various FFN layers, which is evaluated on the dev sets of the three benchmark datasets. Bold values denote the best results.

Model	WNUT2016 (F1-Score)	CoNLL2004 (F1-Score)	SciERC (F1-Score)
S-NER +			
Decoder with 1 FFN layer	**53.77**	87.36	**68.87**
Decoder with 2 FFN layers	51.66	87.54	68.12
Decoder with 3 FFN layers	51.79	**88.71**	67.49

**Table 7:** Performance compared to that of various approaches for obtaining span representation, evaluated on the dev sets of the three benchmark datasets. Bold values denote the best results.

Model	WNUT2016 (F1-Score)	CoNLL2004 (F1-Score)	SciERC (F1-Score)
S-NER +			
Max-pooling	**53.77**	**87.36**	**68.87**
Average-pooling	52.71	86.45	67.43
Boundary	52.23	87.21	66.99

**Table 8:** Comparisons of the model training speed between S-NER and sequence labeling-based NER models. ↑ denotes that a larger value means a faster speed. Bold values denote the best results.

Model	WNUT16 (Sents/s ↑)	Model	CoNLL2004 (Sents/s ↑)	Model	SciERC (Sents/s ↑)
CL-KL	36	Table-sequence	32	RDANER	37
S-NER	**44**	S-NER	**67**	S-NER	**53**

**Table 9:** Case study of the three benchmarks where the blue font denotes the gold entities located in sentences; the red font denotes cascading label misclassifications; and the green font denotes mistakenly predicted entities. In the three cases, all labels and entities in Case rows are gold, and all entities are correctly predicted by S-NER. Furthermore, the CL-KL, RDANER and Table-sequence are the previous best sequence labeling-based NER models on the three benchmarks, respectively.

WNUT2016		
Case 1	Text	Kern Valley will play the San Diego Jewish Academy tomorrow
	Label	B-SpSt I-SpSt O O O B-GeoLoc I-GeoLoc I-GeoLoc I-GeoLoc O
	Entity	(Kern Valley)${}_{\mathrm{sportsteam}}$; (San Diego Jewish Academy)${}_{\mathrm{geo-loc}}$
CL-KL	Label	B-SpSt I-SpSt O O O B-Loc I-Loc B-Org I-org O
	Entity	(Kern Valley)${}_{\mathrm{sportsteam}}$; (San Diego)${}_{\mathrm{loc}}$; (Jewish Academy)${}_{\mathrm{org}}$
S-NER	Entity	(Kern Valley)${}_{\mathrm{sportsteam}}$; (San Diego Jewish Academy)${}_{\mathrm{geo-loc}}$
SciERC		
Case 2	Text	The model is evaluated on English and Czech newspaper texts
	Label	O B-Genric O O O B-Mat I-Mat I-Mat I-Mat I-Mat
	Entity	(model)${}_{\mathrm{genric}}$; (English and Czech newspaper texts)${}_{\mathrm{material}}$
RDANER	Label	O B-Genric O O O B-Mat O B-Mat I-Mat I-Mat
	Entity	(model)${}_{\mathrm{genric}}$; (English)${}_{\mathrm{material}}$; (Czech newspaper texts)${}_{\mathrm{material}}$
S-NER	Entity	(model)${}_{\mathrm{genric}}$; (English and Czech newspaper texts)${}_{\mathrm{material}}$
CoNLL2004		
Case 3	Text	Paul Fournier, a spokesman for the state Department of Inland Fisheries and Wildlife
	Label	B-Per I-Per O O O O O B-Org I-Org I-Org I-Org I-Org I-Org
	Entity	(Paul Fournier)${}_{\mathrm{per}}$; (Department of Inland Fisheries and Wildlife)${}_{\mathrm{org}}$
Table-sequence	Label	I-Per I-Per O O O O B-Org I-Org O B-Other I-Other I-Other I-Other
	Entity	(Paul Fournier)${}_{\mathrm{per}}$; [state Department]${}_{\mathrm{org}}$; [Inland Fisheries and Wildlife]${}_{\mathrm{other}}$
S-NER	Entity	(Paul Fournier)${}_{\mathrm{per}}$; (Department of Inland Fisheries and Wildlife)${}_{\mathrm{org}}$

## Data Availability

Publicly available datasets were analyzed in this study. The data can be accessed at: https://github.com/lavis-nlp/spert (accessed on 6 August 2021) and https://github.com/cuhksz-nlp/SANER (accessed on 6 August 2021).

## Abbreviations

The following abbreviations are used in this manuscript:

NER	Named Entity Recognition
NLP	Natural Language Processing
S-NER	Span-Based Model for NER
RNN	Recurrent Neural Network
CNN	Convolutional Neural Network
MTL	Multi-Task Learning
CRF	Conditional Random Fields
FFN	Feed-Forward Network
BiLSTM	Bidirectional Long Short-Term Memory
BERT	Bidirectional Encoder Representation from Transformers
